# Civilization in Its Relation to the Decay of Teeth

**Published:** 1882-12

**Authors:** Norman W. Kingsley

**Affiliations:** New York


### Civilization in its Relation to the Decay of the Teeth.

DR. NORMAN W. KINGSLEY, NEW YORK.

[Read before the International Congress, London, 1881.]

The most important undetermined problem now confronting the dental profession is embodied in the inquiry made daily by anxious parents in substantially the following form: "Why do my teeth decay more rapidly than my father's or mother's did, and why are my children's teeth decaying at an earlier age than mine?

This inquiry does not come from those who neglect their teeth and who never consult a dentist until a throbbing pain betrays an exposed nerve. It does not come from the lower classes of society, the ignorant, or the depraved, who are as habitually careless of their teeth as they are of every other personal attention.

It is not confined to any race or nationality, nor is it affected by the seasons, climates, or locality; but it does come from a class which is the most intelligent, the most cultured, and the most finely organized in any community, irrespective of race, locality, or climate, from people who appreciate the importance of hygienic laws, who give much care to the preservation of their bodily health, and who are attentive to their teeth.

This inquiry, when put to the professional adviser, frequently betrays great anxiety for the present condition, and alarm for the final results. It is useless to treat it lightly, or attempt a denial of the premises; for although the inquirer may have no statistics to sustain his impressions, he has a knowledge and a memory which is almost equivalent to a record, and his opinions are confirmed by the observations of most of his acquaintances.

The cases are exceedingly rare, if they exist at all, where the teeth of the children are sounder than the parents', and we must admit the conclusion that with each succeeding generation the dental organs are becoming more and more degenerate.

The dental practitioner holds the position of one in authority to his patients, if he be anything more than a mechanic, or makes any pretension to scientific attainments; but what response has he to their inquiries?

It is not difficult to formulate an answer that will satisfy many. It requires but little tact, and less knowledge, to give a reply that appears to impart information, but which really only covers ignorance and pacifies the patient. Even those who are well versed in other branches of science are often satisfied with an answer that will not bear investigation, and may even be absurd.

In this way is heard daily a repetition of the theories and speculations of a generation which have just enough foundation in truth and just enough plausibility to escape being challenged.

Probably the most universal idea among cultivated unprofessional people of the cause of the decay of teeth, especially among children, is "candy." At least, nine-tenths of parents seem to think there is no other cause, and half believe that, with all their efforts to keep candy away from their children, they must be eating it surreptitiously, or their teeth would not be found decayed.

There is probably no more fallacious idea so generally believed. How it should have obtained such wide-spread credence is difficult to conceive; it has just sufficient plausibility to save it from rejection.

But pure candy, in moderate quantity, never harmed any healthy child or adult. Pure candy, which is only sugar in a fanciful form, is as legitimate an article of diet, within moderate limits, as pastries, pickles or preserves, or a hundred other inventions for gratifying the palate, and if all other factors were eliminated from the problem, candy might be eaten with impunity.

Another speculation which has gained some credence is contained in the discovery that ice water, hot drinks, or hot cakes are the cause of all the mischief. It was evenly gravely asserted, at a gathering of professional men a few years since, by a gentleman who had no mean pretensions to scientific acquirements, that the common habit among Americans of drinking hot coffee or tea, to be followed by ice water, caused a sudden alternate expansion and contraction of the enamel, which produced cracks, which ultimated in cavities of decay. It is very doubtful if a cavity of decay ever originated from that cause, and certainly it is not possible that such a cause can have any very considerable direct influence. Neither ice-water nor hot drinks are any perversion of nature's provision for man's health or comfort.

If there are any pernicious results following their use, from which the teeth suffer, it is not in their directly causing caries or other injury to these organs.

Another favorite theory held by some professional gentlemen is that of "living upon soft food instead of that which requires mastication." It has a seeming plausibility from the fact that the principal tissues of the body become strengthened by the severity of their use, and are atrophied by neglect. But among the opposing arguments to this theory may be noted the fact that the Esquimaux living on whale blubber, the tropical Oriental with a rice diet, and the savage with his forest nuts, grains and dried meats, have each and all equally good masticating organs—the difference appearing not in structure, nor liability to decay, but in the abrasion of those which have the most severe use. Food has unquestionably a most important influence upon the dental organs, but the benefits come not in the rejection of soft food and the substitution of hard. Neither is the "bran-bread"

theory, which has been so persistently advanced, a much more solid foundation upon which to rest.

Bread made from unbolted flour, or that which contains the husk of the grain, possesses certainly more of the elements which go to make up healthy tooth-structure than where these elements have been eliminated. But mankind do not live upon bread diet alone, it forms but a small part of the bill of fare of the more luxurious classes, and it is very doubtful if the experiment were tried of feeding a family from birth to maturity upon bran bread, they would show any marked improvement in the dental organs, if that were the only means relied upon to produce that result. It savours of the experiment of feeding phosphates into the stomach, in the expectation of thereby making solid bones.

The food theories may be among the secondary sources of decay, but no one of them, nor all combined, form the primary cause of the present increasing degeneration of tooth-structure.

Still another theory persistently advocated by a professional observer, is embodied in the statement that "Contact always produces decay." That teeth frequently decay when they are in contact must be admitted; that they are more likely to decay at such points than in more exposed portions, is also borne out by every observer; but that contact is anything more than an incident to the real cause cannot be shown. If contact be a cause operating independently of other causes, then are we confronted with the fact that a different, but equally independent and potent cause, is found operating upon another surface of the tooth where there is no contact; and, furthermore, an examination of the most solid dental structures ever presented for professional inspection, show all the teeth in absolute contact, and no evidence of decay. This condition is also universally found in the jaws of savage races of good physique.

The contact theory must be relegated to its proper place among secondary causes, and simply a coincident factor in the great problem.

Another theory which has been ably maintained as the sole cause of such vast destruction of dental organs, is the want of cleanliness.

This proposition becomes more forcible when put in the affirmative form—viz., that if the teeth are kept absolutely clean they will not decay.

It is not probable that so simple and so direct a statement, so broad, so comprehensive, and so complete, can be made embodying any other idea, or the different ideas which have been advanced.

It is upon the face of it unquestionably true, and would seem to prove that the converse statment of want of cleanliness was the real cause underlying tooth degeneration. But this is not true. Want of cleanliness is only a factor associated with many others to give it any potency. With all other factors eliminated, it is not at all likely that teeth would ever decay, simply because they were not cleaned.

The cleaning of teeth, as it is now conducted with lotions and pastes, with powders, brushes, and silk, was unknown to the ancients, who were innocent of any thought of such a necessity, and besides we are even now frequently seeing perfect dental structures, which have never known cleanliness as at present understood.

Should we accept want of cleanliness as the sufficient answer to our inquiries, it would dispose of the necessity of any discussion of the agency of "Electrical Conditions," "Chemical Solutions," "Acid Secretions," "Fungi" or "Parasites," by whatever name they might be called; for whatever might be the agency, external and foreign to tooth substances, which was acting detrimentally, absolute cleanliness would prove the radical cure. Nevertheless, this most plausible of all practical theories is more important in its remedial character than as an explanation of the primary cause.

Each and all the theories thus far noted may be classed among secondary causes, any one of which may have some direct application in certain individual cases, but neither one, nor altogether reach the root and foundation of the difficulty.

"Climate influences" is the cause, says another, and still another, "Intermarriage, or the Mixing of Types." These terms, though not altogether meaningless, have probably, in the minds of those who use them, only a vague and indefinite relation to the subject. The more one reflects upon it the less is he inclined to adopt the idea that climatic conditions are anything but a coincidence.

Climate, *per se* cannot, possibly influence directly the disorganization of tooth structure; the changes from heat to cold, from damp to dry, from high barometer to low, do not in themselves affect tooth substance, no matter how frequent or how sudden. Nor does the prolonged continuance of any one of these conditions, with or without any of the others, produce any visible change. The inhabitants of the torrid and the frigid zones have equally sound masticating organs, and so have those of high altitudes, under a prevailing low barometer, as well as those at the sea-side, under the other extreme.

Districts may be malarious to the unacclimatized, and the whole system poisoned, but tooth-structure suffers, if at all, only as a secondary result.

Inter-marriage, inter-breeding, or crossing the different types of the human race, ought not to result in defective tooth structure. Reasoning from analogy, in the crossing of different breeds of animals improvement should be looked for and not degeneration.

Inter-breeding among diseased, deformed, or unsound subjects, carries with it its own antidote, and would very shortly exterminate the race, but the fault would not lie with the principle involved, but with the subject chosen. Inter-breeding, in its highest application and essence, involves that only sound and healthy subjects be brought together, and a superior quality, rather than inferior, is the natural result.

While this applies to structure, it may not be equally true of arrangemeut. The mixture of sound, but inharmonious, types, may result in deformity of arrangement, but not in disorganization or deterioration of structure.

Inter-breeding of unsound and diseased subjects with the sound and healthy, is equally a perversion of and an abuse of the principle.

Other vague theories—used, often, without much understanding, but of general application—are formulated as "Constitutional Conditions," "Violations of Hygienic Laws," or "Hereditary Predisposition;" but the most comprehensive of all agencies, and the most intangible withal, which has been made sponsor for this growing curse of the human race, is—"Civilization." What is civilization?—and what has civilization to do with decaying teeth?

Civilization means—out of barbarism, into refinement; out of ignorance, into knowledge; out of bondage, into liberty; out of privation, into comfort.

Civilization expands the intellect, represses vice and savage instincts, cultivates virtue and noble aspirations, encourages the growth of the emotional nature, and enlarges the domain of human sympathy. Civilization includes a life of luxury and ease, the accumulation of wealth, the development of the arts, and the gratification of the appetites and the æsthetic tastes. Civilization defies and controls the elements, organizes commerce, builds cities, railways, telegraphs, and factories, and brings to our vision the most infinitesimal organisms, as well as the mighty worlds of boundless space.

Civilization is the divinely-appointed method through which mankind derive their greatest blessings, and by which they reach their highest possible state of intellectual, moral, and social development. Through civilization is to come the grandest achievements of the race, the possibilities of which are so much greater than have even been conceived, that if now presented to the mind they would be regarded as proceeding only from the Infinite; and only through civilization will the millennial, or ideal, existence of the human race ever be attained.

Civilization, therefore, is a normal condition of mankind, entirely consistent with perfect health and development, and is the agency of evolution from a lower to a higher grade. Nevertheless, while civilization thus contains within itself the elements of the highest attainable finite perfection, it is made the author of nine-tents of the disorders which now afflict mankind. In the rude and barbaric state disease is as uncommon as among the brutes; the sickly and feeble must go to the wall; the fittest only can survive. But with refinement and culture comes enlargement of human sympathy, and the weak and suffering are cherished with fostering care; the sickly child is nurtured tenderly and carefully into manhood, to become the progenitor of a tainted family, and the seeds are sown which bear fruit for generations.

Civilization is not responsible for the disorders of civilized communities; but disease, deterioration, degeneration, and decay, arise from the neglect or abuse, of the resources, the agencies, and the products which civilization brings. Crowded commumities, filthy abodes, vitiated atmosphere, hunger, privation, and exposure—among the lower classes—breed diseases which sap the foundations of life. But neither of these, nor all combined, tend to produce the disastrous effects which follow the habits and mode of life of the more intelligent, the more wealthy, and the more cultured classes.

The increasing tendency to the degeneration of the dental organs of these days and which has now become alarming, is exceptional among the lower classes, while they remain in that class; it becomes distinctly marked when, by the accumulation of wealth, they are enabled to adopt the practices of the higher classes, and thus become subject to the same deteriorating influences.

Caries of the teeth is only incidentally connected with the diseases which prevail among a class inured to constant physical labor, whose minds are fully occupied in solving the question of daily bread, and who are content when food, clothing, and shelter are provided. Fevers, inflammations, or epidemic diseases, do not, necessarily, cause teeth to decay. People are constantly passing through these various complaints, and the teeth remain intact. But, passing out of this condition in life, into the more refined and luxurious, we find physical labor exchanged for mental; strain of mind takes the place of strain of body; muscular tension ceases, and nervous tension takes its place; the vices of luxury supplant the virtues of privation; the individual, no longer content with having satisfied his daily wants, enters the struggle to obtain a higher place in the social scale, to accumulate wealth, and to be envied by his former associates.

Refinement and culture create new ambitions, new responsibilities, and new anxieties. Every added anxiety brings an additional mental strain. The mind—and the mind only—is constantly on the alert. The brain has no rest. Nutrition of muscles and bones is diverted to repair the undue waste of nervous tissue; and sooner or later come, inevitably, to parent or offspring, the long list of nervous diseases which now so threateningly confront us. The testimony of the most distinguished neurologists—especially in America—is uniform and undeniable, that diseases of the brain, the spinal cord, and the nerves which issue from them, or the sympathetic system, are alarmingly on the increase. The causes which tend directly to produce such results are more potent, and more active, in the Northern United States than in any other locality upon the face of the globe.

Causes and results which might pass unnoticed in other localities, from their infrequency, are there exhibited in their most pronounced forms; and thus better opportunities are there afforded for the study of these phenomena than anywhere else. The reasons for this (which time will not permit us to discuss) have their foundations, primarily, in the institutions of the country, which permit every one to indulge his wildest political and social ambitions. Add to this, opportunities for accumulating wealth unparalleled in the history of nations, together with the influence of railways, steam printing-presses, and telegraphs—which bring a whole nation into a common bond of sympathy—and we find every excitement, and every stimulus, for the intensest mental activity. For these reasons, causes multiply and results develop with far greater rapidity in the New World than in the old; but testimony is not wanting, that the same thing, in kind, but more limited in extent, is now going on in Great Britain; and even heretofore stolid Germany, is developing a like transition.

Decaying teeth and nervous diseases are correlated—each, in a measure, resulting from the other, and both symptoms of a common cause. Neurasthenia, or nervous exhaustion, is the almost uninterrupted condition of large numbers of the most highly cultivated and most charming people of modern society. I use the term "nervous exhaustion," not in a positive, but in a relative, sense. Thousands, among the classes referred to, may not show the positive symptoms of nervous exhaustion, but they have, nevertheless, no reserve nervous force.

With watchfulness and attention to their health, and from long habit in a beaten track, they may be quite equal to a systematized daily routine, but any undue excitement, any excessive emotional strain, any unusual physical labor, becomes a drain upon a nervous system without a reserve force which breaks it down, carrying it beyond the point or possibility of immediate recuperation.

Every intellectual faculty developed by civilization, every emotional excitement of a finely strung nervous organization, contains within it the possibilities of the highest enjoyment, but when stimulated to excess or abnormally used, impairs with unerring certainty other bodily functions.

The teeth require as constant nutrition as the muscles, the bones, or any other organs or ti-sues of the system.

Teeth decay, primari y, because the nutrition of their organic structures being withdrawn, retrograde metamorphosis ensues; and, secondarily, because the agencies born of diverted, improper, or inadequate nutrition, are capable of producing their chemical solution.

Caries is simply solution or disorganization of tooth constituents by agents which are always external, but which would be quite inert under other constitutional conditions.

The vitality of well organized tooth-structure in a healthy body is quite sufficient to counteract the effects of any active external agents which might be temporarily present; but when nutrition is insufficient or diverted, the resisting power of its vitality inadequate, and destructive agents present, the teeth will yield at their weakest points, and caries result.

Herein we find the explanation of many cases which are seen of caries having made considerable progress and then ceased, the causes which produced it having evidently spent their force, or rather the conditions which permitted caries to advance having terminated.

Parents whose teeth are decaying because of diverted nutrition, and exhausted nervous force, transmit, by the inevitable laws of inheritance, this condition to their offspring and it becomes in them constitutional.

Children born of parents whose physical health is barely equal to a continuance of their mental activities, enter the life-race handi-capped.

That which was possibly but a temporary state in the parents, which might pass of itself, leaving no trace, or, at least, without serious effects upon the teeth, may become the fixed and permanent characteristic of the child, to be fought against and combated through life.

A child entering life under such unpromising and predisposing circumstances, will almost inevitably show frailty of physique, with poorly organized teeth and a certainty of their early decay.

Unless active and constant effort is made to arrest the result, the steps on the downward grade are as inevitable as mathematical law. Every mental or emotional excitement, every intellectual stimulus in early childhood, only aggravates the inherited and constitutional tendency, and the teeth succumb to destructive agencies which would otherwise be harmless.

The physiological law and its rationale are very simple. Excessive mental or emotional activity impairs digestion and diverts nutrition to the brain to repair undue waste; from lack of nutrition the teeth become degraded, and fall an easy prey to the aggressive agencies generated by the same disordered and defective assimilation.

The organic condition of the tooth, resulting from the want of proper nutrition, is thus the primary cause or essential element, either directly or originally, which gives potency to other causes which are superficial.

Given an unimpaired nervous system, in a sound body without inherited taint, and teeth would never decay so long as that condition was maintained, and that, too, irrespective of climate, peculiar kinds of food, or any external agents which might be present in the natural course of nutrition. Even cleanliness, now considered so essential, would be unnecessary as a preservative of the teeth, and need be practiced only for its comfort. We thus recognize the relation and the influence of the many secondary causes, which, by one or another, have been invested with such importance.

The radical remedy for these evils, and the salvation of the race from degeneracy and destruction, would seem to involve a return to a habit of life more consistent with hygienic laws, but in the present irresistable march of civilization, a return to a primitive condition of life would be impossible; but a recognition of, and an adaptation to, a new condition of society is not only possible, but practicable.

A pessimistic view of the future is without reason, for while degeneracy of the teeth is certainly on the increase with certain families and classes, there are equally certain signs of its abatement. With increasing wealth in families comes less care, less anxiety, less nervous strain, more ease, more attention to hygiene, and better habits of life. Destruction of dental organs is arrested in childhood, and transmission of tendency is to a considerable extent stamped out.

The intellectual activity of to-day, the driving energy and intensity of modern thought, is not inconsistent with sound constitutions, with perfect health in all the tissues, and with long life. It is the worry which wears out the nervous system, and not the work.

Civilization, the glory of mankind in their maturity—which has rapidly and largely advanced at the expense of the best life-blood of the race, is, nevertheless, in no wise responsible for the incidental effects which have resulted from a violation of her true principles, for out of civilization ought to, and must come, the grandest examples of physical beauty the world has yet seen, without spot or blemish or taint of disease.

In Providence, R. I., with a population of 104,000, not a single death has occurred from smallpox since 1875. The reason is given as "general and careful vacinnation."

It is the opinion of the most experienced health authorities in London that the poorer classes of Jews are naturally long lived, and that the dietary and other sanitary regulations prescribed by their religion enable them to battle for a considerable time against unhealthy surroundings.

